# Supplementary material for: Resolving the full spectrum of human genome variation using Linked-Reads
Source: Genome Res. 2019 Apr;29(4):635–45. doi: 10.1101/gr.234443.118 (PMC6442396; doi:10.1101/gr.234443.118)
Supplement: Supplemental Material [file supp_gr.234443.118_Supplemental_longranger-2.2.zip › longranger-2.2.tar.bz2!/lr2.2-public/lib/stan/cmdstan/stan/doc/api/html/transform_8hpp.html]

Stan: src/stan/prob/transform.hpp File Reference


|  |  |
| --- | --- |
|  | Stan  2.5.0  probability, sampling & optimization |


- Main Page
- Related Pages
- Namespaces
- Classes
- Files

- File List
- File Members

All Classes Namespaces Files Functions Variables Typedefs Enumerations Enumerator Friends Macros Pages

- src
- stan
- prob

Namespaces |
Functions |
Variables

transform.hpp File Reference

`#include <cmath>`  
`#include <cstddef>`  
`#include <limits>`  
`#include <stdexcept>`  
`#include <sstream>`  
`#include <vector>`  
`#include <boost/throw_exception.hpp>`  
`#include <boost/math/tools/promotion.hpp>`  
`#include <stan/agrad/rev/matrix.hpp>`  
`#include <stan/math.hpp>`  
`#include <stan/math/error_handling.hpp>`  
`#include <stan/math/error_handling/matrix/check_square.hpp>`  
`#include <stan/math/matrix.hpp>`  
`#include <stan/math/matrix/sum.hpp>`  
`#include <stan/math/matrix_error_handling.hpp>`  
`#include <stan/math/matrix/multiply_lower_tri_self_transpose.hpp>`  
`#include <stan/math/matrix/meta/index_type.hpp>`

Go to the source code of this file.

|  |  |
| --- | --- |
| Namespaces | |
|  | stan |
|  | Probability, optimization and sampling library. |
|  | |
|  | stan::prob |
|  | Templated probability distributions. |
|  | |

|  |  |
| --- | --- |
| Functions | |
| template<typename T > | |
| void | stan::prob::factor\_U (const Eigen::Matrix< T, Eigen::Dynamic, Eigen::Dynamic > &U, Eigen::Array< T, Eigen::Dynamic, 1 > &CPCs) |
|  | This function is intended to make starting values, given a unit upper-triangular matrix U such that U'DU is a correlation matrix. More... |
|  | |
| template<typename T > | |
| bool | stan::prob::factor\_cov\_matrix (const Eigen::Matrix< T, Eigen::Dynamic, Eigen::Dynamic > &Sigma, Eigen::Array< T, Eigen::Dynamic, 1 > &CPCs, Eigen::Array< T, Eigen::Dynamic, 1 > &sds) |
|  | This function is intended to make starting values, given a covariance matrix Sigma. More... |
|  | |
| template<typename T > | |
| Eigen::Matrix< T,   Eigen::Dynamic, Eigen::Dynamic > | stan::prob::read\_corr\_L (const Eigen::Array< T, Eigen::Dynamic, 1 > &CPCs, const size\_t K) |
|  | Return the Cholesky factor of the correlation matrix of the specified dimensionality corresponding to the specified canonical partial correlations. More... |
|  | |
| template<typename T > | |
| Eigen::Matrix< T,   Eigen::Dynamic, Eigen::Dynamic > | stan::prob::read\_corr\_matrix (const Eigen::Array< T, Eigen::Dynamic, 1 > &CPCs, const size\_t K) |
|  | Return the correlation matrix of the specified dimensionality corresponding to the specified canonical partial correlations. More... |
|  | |
| template<typename T > | |
| Eigen::Matrix< T,   Eigen::Dynamic, Eigen::Dynamic > | stan::prob::read\_corr\_L (const Eigen::Array< T, Eigen::Dynamic, 1 > &CPCs, const size\_t K, T &log\_prob) |
|  | Return the Cholesky factor of the correlation matrix of the specified dimensionality corresponding to the specified canonical partial correlations, incrementing the specified scalar reference with the log absolute determinant of the Jacobian of the transformation. More... |
|  | |
| template<typename T > | |
| Eigen::Matrix< T,   Eigen::Dynamic, Eigen::Dynamic > | stan::prob::read\_corr\_matrix (const Eigen::Array< T, Eigen::Dynamic, 1 > &CPCs, const size\_t K, T &log\_prob) |
|  | Return the correlation matrix of the specified dimensionality corresponding to the specified canonical partial correlations, incrementing the specified scalar reference with the log absolute determinant of the Jacobian of the transformation. More... |
|  | |
| template<typename T > | |
| Eigen::Matrix< T,   Eigen::Dynamic, Eigen::Dynamic > | stan::prob::read\_cov\_L (const Eigen::Array< T, Eigen::Dynamic, 1 > &CPCs, const Eigen::Array< T, Eigen::Dynamic, 1 > &sds, T &log\_prob) |
|  | This is the function that should be called prior to evaluating the density of any elliptical distribution. More... |
|  | |
| template<typename T > | |
| Eigen::Matrix< T,   Eigen::Dynamic, Eigen::Dynamic > | stan::prob::read\_cov\_matrix (const Eigen::Array< T, Eigen::Dynamic, 1 > &CPCs, const Eigen::Array< T, Eigen::Dynamic, 1 > &sds, T &log\_prob) |
|  | A generally worse alternative to call prior to evaluating the density of an elliptical distribution. More... |
|  | |
| template<typename T > | |
| Eigen::Matrix< T,   Eigen::Dynamic, Eigen::Dynamic > | stan::prob::read\_cov\_matrix (const Eigen::Array< T, Eigen::Dynamic, 1 > &CPCs, const Eigen::Array< T, Eigen::Dynamic, 1 > &sds) |
|  | Builds a covariance matrix from CPCs and standard deviations. More... |
|  | |
| template<typename T > | |
| const Eigen::Array< T,   Eigen::Dynamic, 1 > | stan::prob::make\_nu (const T eta, const size\_t K) |
|  | This function calculates the degrees of freedom for the t distribution that corresponds to the shape parameter in the Lewandowski et. More... |
|  | |
| template<typename T > | |
| T | stan::prob::identity\_constrain (T x) |
|  | Returns the result of applying the identity constraint transform to the input. More... |
|  | |
| template<typename T > | |
| T | stan::prob::identity\_constrain (const T x, T &) |
|  | Returns the result of applying the identity constraint transform to the input and increments the log probability reference with the log absolute Jacobian determinant. More... |
|  | |
| template<typename T > | |
| T | stan::prob::identity\_free (const T y) |
|  | Returns the result of applying the inverse of the identity constraint transform to the input. More... |
|  | |
| template<typename T > | |
| T | stan::prob::positive\_constrain (const T x) |
|  | Return the positive value for the specified unconstrained input. More... |
|  | |
| template<typename T > | |
| T | stan::prob::positive\_constrain (const T x, T &lp) |
|  | Return the positive value for the specified unconstrained input, incrementing the scalar reference with the log absolute Jacobian determinant. More... |
|  | |
| template<typename T > | |
| T | stan::prob::positive\_free (const T y) |
|  | Return the unconstrained value corresponding to the specified positive-constrained value. More... |
|  | |
| template<typename T , typename TL > | |
| T | stan::prob::lb\_constrain (const T x, const TL lb) |
|  | Return the lower-bounded value for the specified unconstrained input and specified lower bound. More... |
|  | |
| template<typename T , typename TL > | |
| boost::math::tools::promote\_args  < T, TL >::type | stan::prob::lb\_constrain (const T x, const TL lb, T &lp) |
|  | Return the lower-bounded value for the speicifed unconstrained input and specified lower bound, incrementing the specified reference with the log absolute Jacobian determinant of the transform. More... |
|  | |
| template<typename T , typename TL > | |
| boost::math::tools::promote\_args  < T, TL >::type | stan::prob::lb\_free (const T y, const TL lb) |
|  | Return the unconstrained value that produces the specified lower-bound constrained value. More... |
|  | |
| template<typename T , typename TU > | |
| boost::math::tools::promote\_args  < T, TU >::type | stan::prob::ub\_constrain (const T x, const TU ub) |
|  | Return the upper-bounded value for the specified unconstrained scalar and upper bound. More... |
|  | |
| template<typename T , typename TU > | |
| boost::math::tools::promote\_args  < T, TU >::type | stan::prob::ub\_constrain (const T x, const TU ub, T &lp) |
|  | Return the upper-bounded value for the specified unconstrained scalar and upper bound and increment the specified log probability reference with the log absolute Jacobian determinant of the transform. More... |
|  | |
| template<typename T , typename TU > | |
| boost::math::tools::promote\_args  < T, TU >::type | stan::prob::ub\_free (const T y, const TU ub) |
|  | Return the free scalar that corresponds to the specified upper-bounded value with respect to the specified upper bound. More... |
|  | |
| template<typename T , typename TL , typename TU > | |
| boost::math::tools::promote\_args  < T, TL, TU >::type | stan::prob::lub\_constrain (const T x, TL lb, TU ub) |
|  | Return the lower- and upper-bounded scalar derived by transforming the specified free scalar given the specified lower and upper bounds. More... |
|  | |
| template<typename T , typename TL , typename TU > | |
| boost::math::tools::promote\_args  < T, TL, TU >::type | stan::prob::lub\_constrain (const T x, const TL lb, const TU ub, T &lp) |
|  | Return the lower- and upper-bounded scalar derived by transforming the specified free scalar given the specified lower and upper bounds and increment the specified log probability with the log absolute Jacobian determinant. More... |
|  | |
| template<typename T , typename TL , typename TU > | |
| boost::math::tools::promote\_args  < T, TL, TU >::type | stan::prob::lub\_free (const T y, TL lb, TU ub) |
|  | Return the unconstrained scalar that transforms to the specified lower- and upper-bounded scalar given the specified bounds. More... |
|  | |
| template<typename T > | |
| T | stan::prob::prob\_constrain (const T x) |
|  | Return a probability value constrained to fall between 0 and 1 (inclusive) for the specified free scalar. More... |
|  | |
| template<typename T > | |
| T | stan::prob::prob\_constrain (const T x, T &lp) |
|  | Return a probability value constrained to fall between 0 and 1 (inclusive) for the specified free scalar and increment the specified log probability reference with the log absolute Jacobian determinant of the transform. More... |
|  | |
| template<typename T > | |
| T | stan::prob::prob\_free (const T y) |
|  | Return the free scalar that when transformed to a probability produces the specified scalar. More... |
|  | |
| template<typename T > | |
| T | stan::prob::corr\_constrain (const T x) |
|  | Return the result of transforming the specified scalar to have a valid correlation value between -1 and 1 (inclusive). More... |
|  | |
| template<typename T > | |
| T | stan::prob::corr\_constrain (const T x, T &lp) |
|  | Return the result of transforming the specified scalar to have a valid correlation value between -1 and 1 (inclusive). More... |
|  | |
| template<typename T > | |
| T | stan::prob::corr\_free (const T y) |
|  | Return the unconstrained scalar that when transformed to a valid correlation produces the specified value. More... |
|  | |
| template<typename T > | |
| Eigen::Matrix< T,   Eigen::Dynamic, 1 > | stan::prob::unit\_vector\_constrain (const Eigen::Matrix< T, Eigen::Dynamic, 1 > &y) |
|  | Return the unit length vector corresponding to the free vector y. More... |
|  | |
| template<typename T > | |
| Eigen::Matrix< T,   Eigen::Dynamic, 1 > | stan::prob::unit\_vector\_constrain (const Eigen::Matrix< T, Eigen::Dynamic, 1 > &y, T &lp) |
|  | Return the unit length vector corresponding to the free vector y. More... |
|  | |
| template<typename T > | |
| Eigen::Matrix< T,   Eigen::Dynamic, 1 > | stan::prob::unit\_vector\_free (const Eigen::Matrix< T, Eigen::Dynamic, 1 > &x) |
|  | |
| template<typename T > | |
| Eigen::Matrix< T,   Eigen::Dynamic, 1 > | stan::prob::simplex\_constrain (const Eigen::Matrix< T, Eigen::Dynamic, 1 > &y) |
|  | Return the simplex corresponding to the specified free vector. More... |
|  | |
| template<typename T > | |
| Eigen::Matrix< T,   Eigen::Dynamic, 1 > | stan::prob::simplex\_constrain (const Eigen::Matrix< T, Eigen::Dynamic, 1 > &y, T &lp) |
|  | Return the simplex corresponding to the specified free vector and increment the specified log probability reference with the log absolute Jacobian determinant of the transform. More... |
|  | |
| template<typename T > | |
| Eigen::Matrix< T,   Eigen::Dynamic, 1 > | stan::prob::simplex\_free (const Eigen::Matrix< T, Eigen::Dynamic, 1 > &x) |
|  | Return an unconstrained vector that when transformed produces the specified simplex. More... |
|  | |
| template<typename T > | |
| Eigen::Matrix< T,   Eigen::Dynamic, 1 > | stan::prob::ordered\_constrain (const Eigen::Matrix< T, Eigen::Dynamic, 1 > &x) |
|  | Return an increasing ordered vector derived from the specified free vector. More... |
|  | |
| template<typename T > | |
| Eigen::Matrix< T,   Eigen::Dynamic, 1 > | stan::prob::ordered\_constrain (const Eigen::Matrix< T, Eigen::Dynamic, 1 > &x, T &lp) |
|  | Return a positive valued, increasing ordered vector derived from the specified free vector and increment the specified log probability reference with the log absolute Jacobian determinant of the transform. More... |
|  | |
| template<typename T > | |
| Eigen::Matrix< T,   Eigen::Dynamic, 1 > | stan::prob::ordered\_free (const Eigen::Matrix< T, Eigen::Dynamic, 1 > &y) |
|  | Return the vector of unconstrained scalars that transform to the specified positive ordered vector. More... |
|  | |
| template<typename T > | |
| Eigen::Matrix< T,   Eigen::Dynamic, 1 > | stan::prob::positive\_ordered\_constrain (const Eigen::Matrix< T, Eigen::Dynamic, 1 > &x) |
|  | Return an increasing positive ordered vector derived from the specified free vector. More... |
|  | |
| template<typename T > | |
| Eigen::Matrix< T,   Eigen::Dynamic, 1 > | stan::prob::positive\_ordered\_constrain (const Eigen::Matrix< T, Eigen::Dynamic, 1 > &x, T &lp) |
|  | Return a positive valued, increasing positive ordered vector derived from the specified free vector and increment the specified log probability reference with the log absolute Jacobian determinant of the transform. More... |
|  | |
| template<typename T > | |
| Eigen::Matrix< T,   Eigen::Dynamic, 1 > | stan::prob::positive\_ordered\_free (const Eigen::Matrix< T, Eigen::Dynamic, 1 > &y) |
|  | Return the vector of unconstrained scalars that transform to the specified positive ordered vector. More... |
|  | |
| template<typename T > | |
| Eigen::Matrix< T,   Eigen::Dynamic, Eigen::Dynamic > | stan::prob::cholesky\_factor\_constrain (const Eigen::Matrix< T, Eigen::Dynamic, 1 > &x, int M, int N) |
|  | Return the Cholesky factor of the specified size read from the specified vector. More... |
|  | |
| template<typename T > | |
| Eigen::Matrix< T,   Eigen::Dynamic, Eigen::Dynamic > | stan::prob::cholesky\_factor\_constrain (const Eigen::Matrix< T, Eigen::Dynamic, 1 > &x, int M, int N, T &lp) |
|  | Return the Cholesky factor of the specified size read from the specified vector and increment the specified log probability reference with the log Jacobian adjustment of the transform. More... |
|  | |
| template<typename T > | |
| Eigen::Matrix< T,   Eigen::Dynamic, 1 > | stan::prob::cholesky\_factor\_free (const Eigen::Matrix< T, Eigen::Dynamic, Eigen::Dynamic > &y) |
|  | Return the unconstrained vector of parameters correspdonding to the specified Cholesky factor. More... |
|  | |
| template<typename T > | |
| Eigen::Matrix< T,   Eigen::Dynamic, Eigen::Dynamic > | stan::prob::cholesky\_corr\_constrain (const Eigen::Matrix< T, Eigen::Dynamic, 1 > &y, int K) |
|  | |
| template<typename T > | |
| Eigen::Matrix< T,   Eigen::Dynamic, Eigen::Dynamic > | stan::prob::cholesky\_corr\_constrain (const Eigen::Matrix< T, Eigen::Dynamic, 1 > &y, int K, T &lp) |
|  | |
| template<typename T > | |
| Eigen::Matrix< T,   Eigen::Dynamic, 1 > | stan::prob::cholesky\_corr\_free (const Eigen::Matrix< T, Eigen::Dynamic, Eigen::Dynamic > &x) |
|  | |
| template<typename T > | |
| Eigen::Matrix< T,   Eigen::Dynamic, Eigen::Dynamic > | stan::prob::corr\_matrix\_constrain (const Eigen::Matrix< T, Eigen::Dynamic, 1 > &x, typename math::index\_type< Eigen::Matrix< T, Eigen::Dynamic, 1 > >::type k) |
|  | Return the correlation matrix of the specified dimensionality derived from the specified vector of unconstrained values. More... |
|  | |
| template<typename T > | |
| Eigen::Matrix< T,   Eigen::Dynamic, Eigen::Dynamic > | stan::prob::corr\_matrix\_constrain (const Eigen::Matrix< T, Eigen::Dynamic, 1 > &x, typename math::index\_type< Eigen::Matrix< T, Eigen::Dynamic, 1 > >::type k, T &lp) |
|  | Return the correlation matrix of the specified dimensionality derived from the specified vector of unconstrained values. More... |
|  | |
| template<typename T > | |
| Eigen::Matrix< T,   Eigen::Dynamic, 1 > | stan::prob::corr\_matrix\_free (const Eigen::Matrix< T, Eigen::Dynamic, Eigen::Dynamic > &y) |
|  | Return the vector of unconstrained partial correlations that define the specified correlation matrix when transformed. More... |
|  | |
| template<typename T > | |
| Eigen::Matrix< T,   Eigen::Dynamic, Eigen::Dynamic > | stan::prob::cov\_matrix\_constrain (const Eigen::Matrix< T, Eigen::Dynamic, 1 > &x, typename math::index\_type< Eigen::Matrix< T, Eigen::Dynamic, 1 > >::type K) |
|  | Return the symmetric, positive-definite matrix of dimensions K by K resulting from transforming the specified finite vector of size K plus (K choose 2). More... |
|  | |
| template<typename T > | |
| Eigen::Matrix< T,   Eigen::Dynamic, Eigen::Dynamic > | stan::prob::cov\_matrix\_constrain (const Eigen::Matrix< T, Eigen::Dynamic, 1 > &x, typename math::index\_type< Eigen::Matrix< T, Eigen::Dynamic, Eigen::Dynamic > >::type K, T &lp) |
|  | Return the symmetric, positive-definite matrix of dimensions K by K resulting from transforming the specified finite vector of size K plus (K choose 2). More... |
|  | |
| template<typename T > | |
| Eigen::Matrix< T,   Eigen::Dynamic, 1 > | stan::prob::cov\_matrix\_free (const Eigen::Matrix< T, Eigen::Dynamic, Eigen::Dynamic > &y) |
|  | The covariance matrix derived from the symmetric view of the lower-triangular view of the K by K specified matrix is freed to return a vector of size K + (K choose 2). More... |
|  | |
| template<typename T > | |
| Eigen::Matrix< T,   Eigen::Dynamic, Eigen::Dynamic > | stan::prob::cov\_matrix\_constrain\_lkj (const Eigen::Matrix< T, Eigen::Dynamic, 1 > &x, size\_t k) |
|  | Return the covariance matrix of the specified dimensionality derived from constraining the specified vector of unconstrained values. More... |
|  | |
| template<typename T > | |
| Eigen::Matrix< T,   Eigen::Dynamic, Eigen::Dynamic > | stan::prob::cov\_matrix\_constrain\_lkj (const Eigen::Matrix< T, Eigen::Dynamic, 1 > &x, size\_t k, T &lp) |
|  | Return the covariance matrix of the specified dimensionality derived from constraining the specified vector of unconstrained values and increment the specified log probability reference with the log absolute Jacobian determinant. More... |
|  | |
| template<typename T > | |
| Eigen::Matrix< T,   Eigen::Dynamic, 1 > | stan::prob::cov\_matrix\_free\_lkj (const Eigen::Matrix< T, Eigen::Dynamic, Eigen::Dynamic > &y) |
|  | Return the vector of unconstrained partial correlations and deviations that transform to the specified covariance matrix. More... |
|  | |

|  |  |
| --- | --- |
| Variables | |
| const double | stan::prob::CONSTRAINT\_TOLERANCE = 1E-8 |
|  | |


---

[ Stan Home Page ]

*© 2011–2014,
Stan Development Team.*
